# The role of social media on COVID-19 preventive behaviors worldwide, systematic review

**DOI:** 10.1371/journal.pone.0306284

**Published:** 2024-07-10

**Authors:** Eneyew Talie Fenta, Eyob Ketema Bogale, Tadele Fentabel Anagaw

**Affiliations:** 1 Department of Public Health, College of Medicine and Health Sciences, Injibara University, Injibara, Ethiopia; 2 Health Promotion and Behavioral Science Department, College of Medicine and Health Science, Bahir Dar, Ethiopia; King Abdulaziz University Faculty of Medicine, SAUDI ARABIA

## Abstract

**Background:**

The likelihood of COVID-19 spreading from one individual to another is impacted by personal factors, preventive actions taken, and the quantity and length of exposure. Social media instantly shares health information with the public so medical professionals can interact with them.

**Methods:**

The review used Preferred Reporting Items for Systematic Reviews and Meta-Analysis (PRISMA) checklist. We retrieve articles by using keywords on Pub Med, Cochrane Library, and grey literatures. The Joanna Briggs Institute (JBI) Meta-Analysis of Statistics Assessment and Review Instrument was used to check the quality of articles. The main qualitative synthesis was including comments related to the characteristics of the studied populations, the study period, and the main results obtained from the social media platforms.

**Result:**

This study includes 32 studies conducted in 20 countries globally. Most of the studies discussed that during the pandemics different types of social medias were utilized to provide knowledge to change the attitude of the people and helps to practices COVID-19 preventive behaviors. By enabling people to seek and share knowledge, socialize, and find pleasure, social media use encourages people to adopt preventive behaviors. This review demonstrated a substantial relationship between higher levels of precautionary behaviors and factors such as educational status, knowledge, fear, and reading medical pages on social media. According to the studies, watching videos is a reliable means to get accurate information, and watching animated films posted on social media can help people learn more about COVID-19 preventive strategies.

**Conclusion:**

In these urgent times, social media could even help with quick information availability; misinformation or inadequate understanding can cause misunderstandings within the community. This analysis revealed that following medical pages on social media, having knowledge, feeling afraid, and having an educational background were all strongly correlated with greater levels of aware preventive behavior. Therefore, it is advised for policy makers to develop social behavioral change health communication strategies, guidelines on COVID-19 prevention behaviors. Health care providers should prepare health learning materials, and provide accurate, updated and timely information using all forms of social media to correct mis- information, misperceptions, depression, and anxiety for better control of the pandemics. Future research shall focus on evaluating effectiveness of each social media platform interventions during such emergency crisis.

## Background

After being originally identified in China in December 2019, the severe acute respiratory syndrome coronavirus 2 disease, or SARS-COV-2, has persisted as a global public health concern. The likelihood of COVID-19 spreading from one individual to another is impacted by personal factors, preventive actions taken, and the quantity and length of exposure [1–3]. When an infected individual speaks, sneezes, coughs, or comes into close contact with another sick person, COVID-19 spreads through secretions, saliva, and respiratory droplets. To avoid the spread of COVID-19, the World Health Organisation (WHO) advises adopting the following preventive measures: staying indoors, covering coughs and sneezes, wearing masks, keeping hands clean, staying a safe distance, taking antiviral medicine, and getting vaccinated. People’s preventive actions during the COVID-19 pandemic determine how well COVID-19 is controlled [4–7].

There were only 20 COVID-19 deaths reported between January 3 and January 9, 2023, despite projections of 300,000 deaths in China up until April 1, 2023, and one million deaths in 2023 [8,9]. At present, the World Health Organization has recorded over 664 million confirmed cases of COVID-19 and over 6.7 million deaths worldwide as of January 24, 2023 [10]. The patients with a history of underlying diseases had a 1.41-fold increased risk of dying compared to those without a history of illness. Additionally, 20.3% of patients disregarded hygiene recommendations, including hand washing, social distancing, and wearing face masks after discharge, and had infected others in their homes [11].

Social media refers to online platforms that enable users to communicate and share material, enabling groups and individuals to interact and exchange information [12,13]. Social media is used to provide immediate health information that health care providers are able to communicate with the community. It is used to facilitate communication between healthcare providers and patients to exchange important health information which is fast, cost-effective, and two-way communication for effective preventive behavioral change [14,15].

The community received their health information primarily from social media. Social media platforms like Twitter, Facebook, YouTube, TikTok, Instagram, and LinkedIn have been utilized to spread public awareness of the COVID-19 epidemic, encourage healthy behavior, and improve community health outcomes using textual content, voice messages, and video clips [16,17]. Social media makes it possible to keep up with current events and provide information on disease-related infection prevention guidelines. Social media health posts offer advice on how to stop the virus from spreading, including how to stay away, wear masks, and wash your hands frequently [16,18–20].

Social media can be used to create virtual communities and deliver news and updates in real time. Two categories of information on the epidemic have garnered public interest on social media. These include detailed case information, such as the movement paths of individual confirmed or suspected patients, their travel history, and the trains or flights they have taken, as well as daily statistical information, such as confirmed cases, new suspected cases, recovered cases, and deaths, both in cumulative numbers and daily updates [21]. Social media is an essential channel for health communication that the general population can use to get online information [7,22,23].

Social media plays a significant role in the rapid exchange of knowledge that leads to preventive actions being taken. In order to encourage users’ preventive actions against behavioral illnesses, social media are useful instruments for health communication. People were said to have been exposed to more COVID-19 information that encourages preventive behaviors when they used social media more frequently and for longer periods of time [20,24–26]. Early in the outbreak, the researchers noted that misinformation about lockdowns and impending government rules was conveyed via text messages. Misinformation regarding misconceptions about the disease is still a concern when it comes to COVID-19 pandemic prevention, despite the efforts of most social media sites to combat it. Effective risk communication for unknown infection threats depends on trust, and behavioral change is influenced by confidence in perceived information [1,27–31]. The vast quantity of COVID-19 information made possible by social media was linked to the public’s perception of COVID-19 susceptibility and severity with increased COVID-19 preventive behaviors. The participants who were exposed to COVID-19 related information on social medias had a positive effect on preventive behaviors [32–34].

During the COVID-19 pandemic, a wider range of information can be shared on social media because to people’s anxiety and willingness to share information, even if they are unsure of it at first [35,36]. The rapid spread of pandemic information on social media has likely raised people’s awareness of the symptoms of COVID-19, the means by which the virus spreads, and the need for preventive measures. However, social media is also rife with false information, rumors, and misconceptions about the illness, which may have contributed to an increase in people’s anxiety, worry, and fear [37,38]. According to the studies, lowering the quantity of false information could reduce the severity of disease. Reducing the transmission of false information or the inclination to believe it could lower the prevalence of infectious diseases. For all diseases, it was anticipated that stage 2 conditions would return to stage 1 after roughly 20% of the population was immunized against false information [39,40]. As a result, a lot of public health organizations disseminated customized information to the public via social media platforms in order to encourage preventative measures and debunk common misconceptions [18,40–43]. Therefore, this systematic review aimed to show the role of social media on COVID-19 preventive behaviors globally.

### Methodology

#### Objective

The primary objective of this review is to synthesize evidence on the role of social media on COVID-19 preventive behaviors globally.

*Review question*. What is the role of social media platforms on COVID-19 preventive behaviors?

#### Searches

This systematic review and meta-analysis were done using Preferred Reporting Items for Systematic Reviews and Meta-Analyses (PRISMA) guidelines. A search strategy was implemented on PubMed, Cochrane Library, and grey literatures, registers, websites, organizations, reference lists and other sources searched or consulted to identify studies, which were systematically searched online to retrieve related articles until June 30/2023 using keywords. (((((((((((social media) OR (Instagram)) OR (YouTube)) OR (Facebook)) OR (Twitter)) OR (TikTok)) OR (LinkedIn)) AND (COVID-19 preventive behavior)) OR (sars-cov-2 preventive behavior)) OR (Coronavirus disease preventive behavior)). We also manually scanned the references of all articles in which full-text reading was performed not to miss additional articles. The review protocol is available on PROSPERO (ID: CRD42023450425).

#### Eligibility criteria

For this review PICO mnemonic (Population, Intervention, control and outcome) approach was used. Participants/population, adult population who uses any form of social media. Outcome: The aim of this study is to synthesize the role of social media on COVID-19 preventive behaviors globally. social media is an important and popular way to disseminate messages to a wide audience, especially those who are frequent users of social media. Intervention, social media are web-based resources or programme that let individuals or communities communicate by sending and receiving text messages, photos, and other types of content. Social networking sites were a part of social media. (e.g., Facebook, YouTube and Instagram), chat or message. Comparator(s)/control, People who adhere COVID-19 preventive behaviors without using social media. This systematic review includes all articles that meet the following criteria: articles that examine the role of social media and COVID-19 preventive behaviors were included. Studies without full text or protocols, conference abstract, books, if they lacked sufficient information on important details of the intervention, articles written in languages other than English, short reports, letters to the editor, and discussions were excluded from the present systematic review.

*Data extraction*. Articles extracted from search engines were exported to a Microsoft Excel spreadsheet after the removal of duplicates. Studies retrieved by using search terms from all databases and additional sources were screened for inclusion criteria. Then, articles that fulfilled the inclusion criteria were undertaken full-text review for admissibility and extraction. The preferred Reporting Item for Systematic Review and Meta-analysis (PRISMA) flowchart was used throughout all steps.

For each included article, the following information was extracted: author, publication year, country, sample size, sampling method, location of data collection, study design, PICOS, follow-up period, citations for each study, measures of COVID-19 preventive behaviors, the role of social media and COVD-19 preventive behaviors and factors associated with social media utilization and COVD-19 preventive behaviors. Titles and abstracts of the studies retrieved were screened independently by two review authors to identify all the articles that potentially meet the inclusion criteria. The full text of the eligible studies was retrieved and independently assessed for eligibility by two review team members. Two researchers performed the data extraction. Disagreements were resolved in consensus or by a third reviewer.

*Quality assessment*. We have used 13-point JBI Critical Appraisal Checklist for Randomized Controlled Trials, for five RCT studies and 8-point JBI Critical Appraisal Checklist for Analytical Cross-Sectional Studies for 27 studies. Using the tool as a protocol, the reviewers (ETF, TDA) used the blinded review approach to evaluate the quality of the original articles. Those studies, scores above the mean for both cross-sectional and interventional studies were considered to have good quality and included in the review. Discrepancies in the quality assessment was resolved through other authors (EKB).

*Strategy for data synthesis*. The extracted data was presented in tables and classified into main topics presenting the main outcomes identified throughout the systematic review process. The main qualitative synthesis was including comments related to the characteristics of the studied populations, the study period, sample size, study design, the role of each social media platforms on COVID 19 preventive behavior adoption. It was impossible to perform a meta-analysis or best evidence narrative synthesis due to the high heterogeneity in the study population, social media platforms, and measuring instruments utilized on study participants to assess social media and COVID-19 preventive behaviors.

## Result

**Fig 1:**
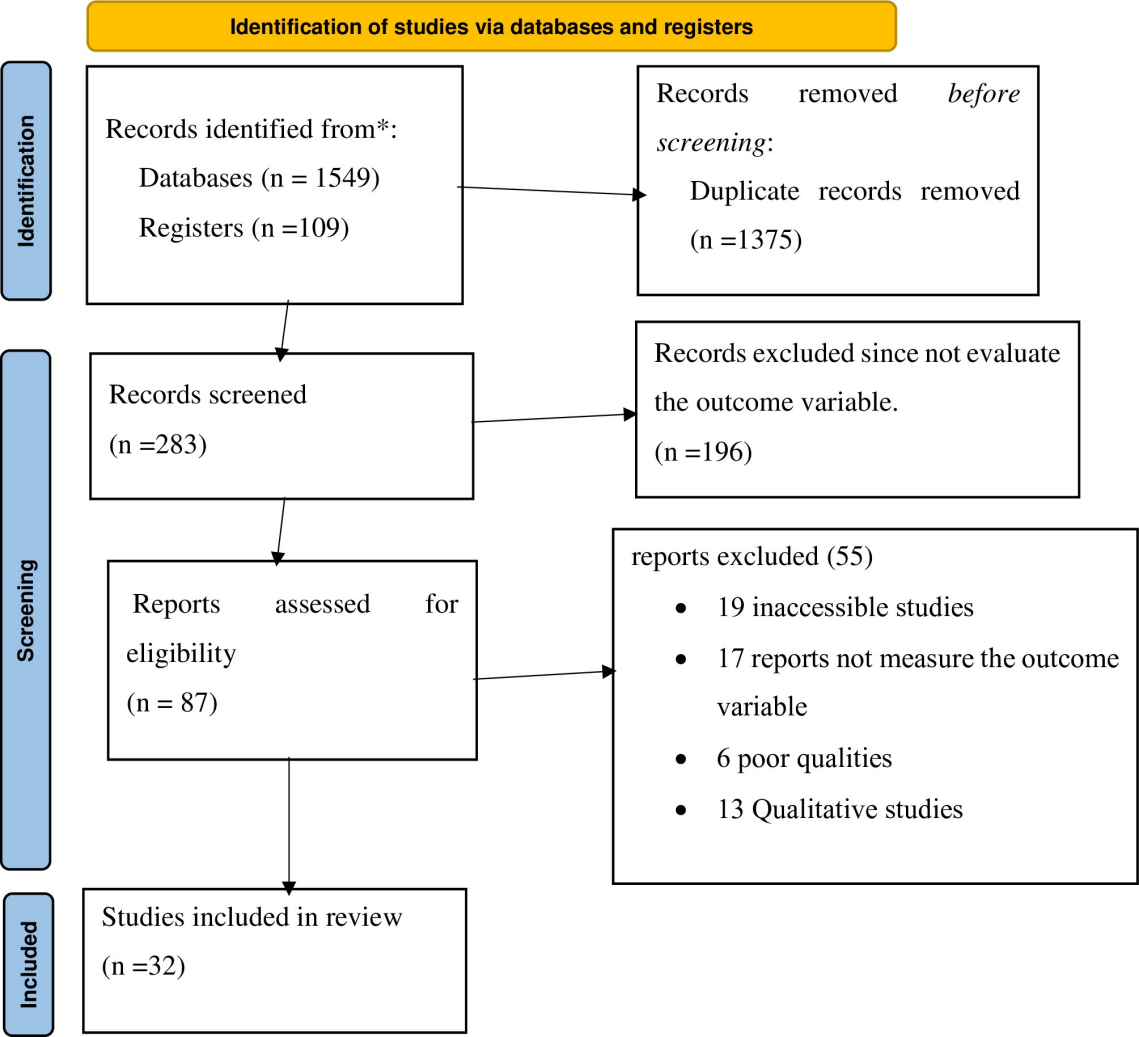
PRISMA flow diagram of study selection for the role of social media on COVID-19 preventive behaviors worldwide, systematic review.

In this systematic review, one thousand six hundred fifty-eight (1658) articles were retrieved from all searched database sources. After the removal of 1375 duplicates, 283 articles were assessed using title and abstract then, 196 articles were excluded since not report the relationship between social media and COVID-19 preventive behaviors. Fifty-four (87) articles were included as shown in Fig 1 in the full-text review,55 articles exclude, and then 32 articles were remained for final systematic review as shown in Fig 1.

### Study characteristics

This study includes 32 studies conducted in 20 countries globally in which, six studies in USA [44–49], China five [34,50–53] Malaysia three,[54–56], Korea two[57,58] Pakistan [59], Spain [60], Poland and Jordan [61], Indonesia [62], Guatemala [63], Japan [64], Jordan [31], Vietnam [65], Bangladesh [66], Ethiopia [67], Italy [68], Nigeria [69], Saudi Arabia [70], Canada [71], SSA [72], and in United States, Mexico, the United Kingdom, Germany, and Spain, [73] one study in each country. Most of the studies were cross-sectional study design except five, which were experimental studies. As explained in Table 1, the total sample size was 37,085 with maximum sample 14,482 sample in USA among experimental studies and minimum of 100 sample size in USA with cross-sectional study (Table 1).

**Table 1:** The role of social media on COVID-19 preventive behaviors worldwide, systematic review.

First author/ Pub year	Study design	Country	Sample size	Social media and COVID-19 preventive behaviors	Quality score
Hernández-García I et al. [60]/2020	cross-sectional study	Spain	129	videos to be viewed in order to obtain reliable information on hand washing practice.	7
Basch CH, et al. [44]/2022	cross-sectional study	USA	100	Wash your hands accounted for 93.3% of the total views. Coverage of the important steps involved in handwashing, such as drying hands, was minimal as was relevant background information.	6
Abuhashesh MY etal.[61]/2021	cross-sectional study	Poland and Jordan	1149	positive relationships between the components of a social media campaign, public health awareness, and behavioral change during COVID-19.	8
Smail EJ et al.[46]/2023	cross-sectional study	USA	1057	Increasing social media use was associated with engagement in more COVID-19 precautionary behaviors.	7
Zhang SX,et al [56]/2020	cross-sectional study	Malaysia	674	More time spent on social media was positively associated with hand washing for males with three or more children. However, for males without children, social media use was negatively associated with 5handwashing.	7
Wardani EM, etal/2020[62]	Experimental	Indonesia	5400	Social media is effectively used to provide health education because it can increase student knowledge and influence behavior in preventing covid-19 transmission.	9
O’Brien N, et al/2022[63]	Experimental	Guatemala	308	GIF exposure significantly improved participants’ self-efficacy, intention and belief on hand washing behavior.	8
Yu J, et al[53] /2022	cross-sectional study	China	122	People who trust in informal information from social media and interpersonal communication would be more likely to adopt mask wearing and hand washing protective behaviors.	5
Suzuki T, et al [64] (2021)	cross-sectional study	Japan	987	social media had stronger predictive power to change attitude through time.	6
Breza E, et al. [45]/2021	Experimental	USA	410	Social media messages recorded by health professionals before the winter holidays in the United States led to a significant reduction in holiday travel and subsequent COVID-19 infections.	6
Yassin A,et al [31](2022)	cross-sectional study	Jordan	827	The variables that were significantly associated with higher levels of precautionary behaviors were knowledge, feeling of fear, and following medical pages on social media.	8
Niu Z, et al/2021[51]	cross-sectional study	China	464	The results suggested that the vulnerable populations’ engagement in coronavirus-related preventive behaviors were significantly associated with barriers, benefits, self-efficacy, trust in doctors’ social media, and trust in TV for COVID-19-related information. Besides, barriers, benefits, self-efficacy, trust in doctors’ social media, and trust in TV mediated the effects of health literacy on preventive behaviors.	7
Duong HT, et al [65]/2021	cross-sectional study	Vietnam	360	Results indicated that interpersonal communication mediated the effect of social media campaign exposure on intentions to stay at home.	7
Tsoy D,et al [49]/2022	cross-sectional study	USA	306	social media would have a positive impact on staying at home intentions. But if users instead perceived messages as being fabricated, amplified, or false, the alert would stay dangerously low.	8
Islam MM,et al [66] /2021	cross-sectional study	Bangladesh	265	Creative social media increase knowledge on COVID-19 prevention measures by using seven online activities which are awareness of Covid-19 spread, wearing the mask, hand washing, precaution before touching the face, nose and eyes, avoiding gathering, social distances, and knowing Covid-19 symptoms. These activities are exhibited in reading essays/writings, seeing photos and flyers, watching videos posting writings, pictures, flyers and videos on social media platforms like Facebook, WhatsApp and YouTube.	5
Wang H,et al[48]/2021	cross-sectional study	USA	500	The excessive information disseminated on social media platforms and other sources is closely related to the dynamics of the general public’s health beliefs.	6
Wu G, et al [52] /2020	cross-sectional study	China	592	Social media information sources, unofficial social media caused both Wuhan and non-Wuhan urban citizens to have higher levels of panic than official media but had no significant impacts on their preventive behaviors.	7
Lu J,et al [34]/2023	cross-sectional study	China	739	Social media empowers individuals in terms of knowledge seeking, knowledge sharing, socializing and entertainment to promote preventive behaviors at the individual level by increasing each person’s perception of collective efficacy and social cohesion.	7
Sadore AA, et al [67]/2021	cross-sectional study	Ethiopia	372	Study participants who had good use of social media to get COVID-19–related information were 9.5 times more engaged in COVID-19 preventive practices than non-users. The study participants who had a high-risk perception of COVID-19 were 2.6 times more engaged in COVID-19 practices compared with study participants who had a low-risk perception of COVID-19.	6
Graffigna G, et al [68]/2020	cross-sectional study	Italy	976	10% of the audience showed an active engagement with the campaign, by expressing likes, by writing comments on, or sharing its contents on Facebook and LinkedIn. Facebook generally appears a more suitable platform for engaging with the audience and as a means to convey public health information in a lively manner.	8
Choi D-H, et al, /2023 [57]	cross-sectional study	Korea	1500	Social media use is positively associated with social norms, which may have a positive relationship with COVID-19 preventive behavior.	6
Mohammed F,[55]/2023	cross-sectional study	Malaysia	488	The findings showed that perceived risk, e-health literacy, public awareness, and health experts’ participation influence public protective behavior when using social media to share COVID-19-relevant content.	5
Okpara CV,et al [69]/2021	cross-sectional study	Nigeria	470	The result showed that recall of messages theme in COVID-19 YouTube animated cartoons significantly predicts health behavior of social media users.	6
Yu S,et al [59] /2022	cross-sectional study	Pakistan	348	The findings approve that attitudes toward social media use in the pandemic have positively mediated the relation between distancing and practices for social media use amid the crisis of COVID-19.	5
Solnick RE, et al [47]/2021	experimental study	USA	2007	The public health messages delivered by physicians and personal messages elicited stronger emotions, greater changes in attitudes, and an increased willingness to disseminate the message than when federal officials delivered impersonal messages.	6
Alrasheed M,et al [70]/2022	cross-sectional study	Saudi Arabia	1500	93.2% of participants use social media for COVID-19 related information. High social media exposure was significantly associated with higher risks of anxiety, depression, and higher levels of COVID-19 risk perception. However, social media has no significant impact on the adoption of preventive behavior.	7
Mat Dawi N,et al /2021[54]	cross-sectional study	Malaysia	404	perception of e-government information and services and perception of social media were found to be significant predictors of attitude toward preventive behavior.	6
Bridgman A,et al [71]/2020	cross-sectional study	Canada	500	Exposure to social media is associated with misperceptions regarding basic facts about COVID-19 while the inverse is true for news media. These misperceptions are in turn associated with lower compliance with social distancing measures.	5
Lee J,et al/2021[58]	cross-sectional study	South Korea	1000	The results reveal that the perceived characteristics of online news and social media influence preventive actions through the trust in citizens or in government.	7
Iyamu I,[72] /2021	cross-sectional study	SSA	1988	The respondents who used social media were more likely to agree that face masks were effective compared with those who did not.	7
Liu PLJSS,et al [50]/2021	cross-sectional study	China	511	Results indicated that personal responsibility partially mediated the relationship between COVID-19 information consumption on social media and preventive behaviors.	6
Vandormael A, et al [73]/2021	RCT	USA, & Europe	14,482	Short, wordless, animated videos, distributed by health authorities via social media, may be an effective pathway for rapid global health communication during health crises.	10

According to a study conducted in Bangladesh, using creative social media to read essays, write articles, viewing flyers and photos, and watch movies increases awareness of COVID-19 preventive actions. These materials can be shared on social media sites like Facebook, WhatsApp, and YouTube. Studies conducted in China found a substantial correlation between trust in doctors’ social media accounts and preventive behaviors related to the coronavirus. Individuals who place greater trust in unofficial information obtained from social media and interpersonal interactions are more likely to wear masks and practice hand washing as preventive measures [34,50–53,66].

Students in Indonesia utilize social media extensively, which has the effect of raising awareness about the prevention of Covid-19 transmission by mask wear, physical distance, and soap washing. In languageless Guatemalan Exposure to graphic interchange formats was statistically linked to improved intentions, self-efficacy, and outcome expectancy beliefs regarding physical distancing; handwashing intentions and outcome expectancy beliefs; and mask-wearing intentions and self-efficacy. The studies discussed individuals who used social media effectively to obtain information about COVID-19 were 9.5 times more likely than non-users to engage in COVID-19 preventive measures [62,63,67].

According to a Korean study, people are more likely to consider preventive behavior if they utilize social media more regularly for news and information regarding COVID-19. When it came to searching for information on COVID-19 pandemic prevention, social media in Japan was more predictive than mass media. Furthermore, following medical pages on social media, feeling afraid, and having knowledge were all strongly linked to higher levels of preventive behavior in Jordan. One particularly effective way to use social media platforms to promote dialogue is by raising public awareness and sensitization about the COVID-19 prevention campaign. Facebook campaigns, for example, have shared information and sparked discussions about pertinent COVID-19 emergency topics between scientists and the general public [57,58,64,68].

Positive correlations were shown between public health awareness, COVID-19 preventative behaviors, and the elements of a social media campaign in studies conducted in Poland and Jordan. Social media is used by 93.2% of participants in Saudi Arabia to obtain information on COVID-19, but it has no discernible effect on the adoption of preventive measures. Social media usage and COVID-19 misperceptions appear to be associated in Canada, indicating that misinformation on social media plays a role in contributing to these misperceptions. People who viewed face masks as useful in avoiding COVID-19 were more likely to utilize social media as their primary source of COVID-19 information, according to a study conducted in six sub-Saharan African nations [61,70,71]. According to research conducted in the United States, TikTok’s general handwashing videos are crucial for educating the public about proper handwashing practices. Emergency physicians are seen to be more successful in disseminating COVID-19 health recommendations when they share their personal stories on Twitter. Intentions to stay at home were influenced by social media campaign exposure, although in Vietnam, this effect was mitigated by interpersonal communication [44–49,65].

Perceived risk, e-health literacy, public awareness, and the involvement of health experts all influence COVID-19 public protective behavior through social media, according to study results from Malaysia. According to a Pakistani study, social media plays a significant impact in social alienation and COVID-19 knowledge. A study conducted in Spain revealed that watching movies is a dependable way to learn about the proper way to wash your hands. In research found in Nigeria, among social media users who watch the COVID-19 YouTube animated cartoon, self-efficacy, task self-efficacy, coping self-efficacy, and outcome expectancy significantly predict health behavior sustainability. According to a study conducted in the US, Mexico, the UK, Germany, and Spain, quick, wordless animated films that are shared on social media by health authorities could be a useful tool for quick worldwide health communication in times of emergency [54–56,59,60,69,73].

## Discussion

In this systematic review 32 articles were included on the role of social media on COVID-19 preventive behaviors globally. Most of the studies discussed that during the pandemics different types of social medias were utilized to provide knowledge to change the attitude of the people and helps to practices COVID-19 preventive behaviors. The use of social media empowers individuals in terms of knowledge seeking, knowledge sharing, socializing and entertainment to promote preventive behaviors. Social media had stronger predictive power to browse information on COVID-19 pandemic prevention [34,46,56,57,61,62,64,67]. This was consistent with the studies discussed that social media platforms played a vital part in controlling pandemics and were useful communication tools for supporting public health initiatives and information dissemination. During the COVID-19 pandemic, social media campaigns were helpful in spreading the word about personal hygiene and social distancing. They also play a significant role in assisting with preparation, reaction, and recovery during medical emergencies [28,30,74–77].

Prior to the US winter holidays, health professionals recorded social media postings that significantly decreased holiday travel and the ensuing COVID-19 infections. Public opinion on health on social media may be somewhat influenced by the dynamics of the pandemic, news, scientific and nonscientific events, and even the relevant tweets that have previously been posted. It is believed that medical professionals who share personal stories on Twitter are more successful at disseminating COVID-19 health recommendations. There was a substantial correlation found between trust in doctors’ social media and preventive behaviors connected to the coronavirus. Medical professionals’ personal and public health messages evoked more powerful feelings, more significant attitude shifts, and more readiness to spread the word than did government authorities’ impersonal remarks [45,47,51]. This was similar to a study that revealed numerous healthcare organizations, physicians, and hospitals have set up Facebook, Twitter, and YouTube accounts to reach out to their patients. Additionally, medical personnel used social media to avoid outbreaks and provide health advice. An increasing number of doctors, scientists, and other health professionals are using Twitter to effectively disseminate information during emergencies and during public health crises in order to communicate suggestions[78–80].

According to this study, social media use was positively correlated with misperceptions about COVID-19, indicating that disinformation on social media may have contributed to some of these misperceptions. Even social media would help people intend to stay at home; if users thought that posts were fake, exaggerated, or manufactured, the alarm level would remain dangerously low [48–50,71]. This was in line with research showing the value of social media in reducing false information, raising public awareness of reliable health-related information, and improving real-time surveillance during pandemics. Even though quick access to information is crucial in these emergency situations, misunderstandings within the community can result from poor comprehension or from erroneous or incorrect information. The COVID-19 pandemic has given rise to more false information on social media, including news that presents two opposing perspectives of the virus. This misinformation affects public safety and makes crisis management more difficult, necessitating health care providers to actively combat both the virus and its associated misinformation at the same time[81–84].

This review demonstrated a substantial correlation between higher levels of precautionary behaviors and educational status, knowledge, fear, and following medical pages on social media. The study also revealed an important connection between the vulnerable populations’ participation in coronavirus-related preventive behaviors and the hurdles, benefits, self-efficacy, and confidence that doctors’ social media accounts provide regarding COVID-19-related information. When compared to study participants who saw COVID-19 as low-risk, those who perceived COVID-19 as high-risk were more involved in COVID-19 behaviors [31,34,44,51,55,62,67]. This study conducted in Uganda and China revealed that individuals with good knowledge engaged in COVID-19 preventative behavior [76,85,86].

There was a strong correlation found between high social media exposure and increased levels of COVID-19 risk perception, anxiety, and depression. Significant determinants of attitude towards preventative behavior were found to be perceptions of social media and e-government information and services. In turn, these misconceptions are linked to a decrease in adherence to social distancing protocols [54,67,70,71]. This has similar finding with the study discussed that dissemination of misinformation about health can lead to unnecessary and undesirable outcomes such as fear, anxiety, misunderstanding of the disease, and problems in the patient-doctor relationship. The studies also reported that social media news may increase anxiety about dangers of the virus, subsequently leading to uptake of precautionary behaviors [19,79,84,87,88].

According to the studies, watching movies is a good way to learn about proper hand washing techniques. Through reading, viewing, and watching films, as well as publishing writings, images, flyers, and videos on social media sites like Facebook, WhatsApp, and YouTube, creative social media raises awareness of COVID-19 preventative methods. During health crises, the animated movies that health officials share on social media could be a useful tool for quick global health communication. The findings demonstrated that social media users’ COVID-19 preventative behavior is significantly predicted by their recollection of the themes from COVID-19 animated cartoons on YouTube [60,63,66,68,69,73]. This was comparable with the study findings reported that videos are attractive and were previously proven to be very effective for health education and behavioral change[15,59,89,90].

### Strengths and limitations

The strength was doing this global systematic review on social media role on COVID 19 preventive behaviors which is the global public health emergency for better utilization of social media and controlling COVID19.its limitation was due to heterogeneity of the study we could not done meta-analysis.

## Conclusion

In this systematic review most of the studies discussed that during the pandemics different types of social medias were utilized to provide knowledge to change the attitude of the people and helps to practices COVID-19 preventive behaviors. Social media helps people to seek and share knowledge, connect with others, and find enjoyment and amusement to support preventive behaviors. When searching for information on COVID-19 pandemic prevention, social media exhibited a better predictive capacity. In these urgent times, social media could even help with quick information availability; misinformation or inadequate understanding can cause misunderstandings within the community.

According to the studies, watching videos is a dependable way to get accurate information, and watching animated films posted on social media can help people learn more about COVID-19 preventive strategies. There was a strong correlation found between high social media exposure and increased levels of COVID-19 risk perception, anxiety, and depression. In turn, these misconceptions are linked to a decrease in adherence to social distancing protocols. This review demonstrated a substantial relationship between higher levels of precautionary behaviors and factors such as educational status, knowledge, fear, and following medical pages on social media. Therefore, it is advised for policy makers to develop social behavioral change health communication strategies, guidelines on COVID-19 prevention behaviors. Health care providers should prepare health learning materials, and provide accurate, updated and timely information using all forms of social media to correct mis- information, misperceptions, depression, and anxiety for better control of the pandemics. Future research shall focus on evaluating effectiveness of each social media platform interventions during such emergency crisis.

## Supporting information

S1 Fig PRISMA flow diagram of study selection ON The role of social media on COVID-19 preventive behaviors worldwide, systematic review. (DOCX)

S1 Table The role of social media on COVID-19 preventive behaviors worldwide, systematic review. (PDF)

## References

[1] Wang C, Pan R, Wan X, Tan Y, Xu L, Ho CS, et al. Immediate psychological responses and associated factors during the initial stage of the 2019 coronavirus disease (COVID-19) epidemic among the general population in China. 2020;17(5):1729. doi: 10.3390/ijerph17051729 32155789 PMC7084952

[2] Wu Z, McGoogan JMJj. Characteristics of and important lessons from the coronavirus disease 2019 (COVID-19) outbreak in China: summary of a report of 72 314 cases from the Chinese Center for Disease Control and Prevention. 2020;323(13):1239–42. 10.1001/jama.2020.2648 32091533

[3] Wang D, Hu B, Hu C, Zhu F, Liu X, Zhang J, et al. Clinical characteristics of 138 hospitalized patients with 2019 novel coronavirus–infected pneumonia in Wuhan, China. 2020;323(11):1061–9. doi: 10.1001/jama.2020.1585 32031570 PMC7042881

[4] Control CfD, Prevention. Overview of testing for SARS-CoV-2 (COVID-19). 2020.

[5] Garcia LPJEeSdS. Use of facemasks to limit COVID-19 transmission. 2020;29. 10.5123/S1679-49742020000200021 32321003

[6] McIntosh K, Hirsch MS, Bloom AJUAohwuccc—e-v-a-p. COVID-19: Epidemiology, virology, and prevention. 2021.

[7] Dai B, Fu D, Meng G, Liu B, Li Q, Liu XJPAR. The effects of governmental and individual predictors on COVID‐19 protective behaviors in China: A path analysis model. 2020;80(5):797–804. 10.1111/puar.13236 PMC7276878 32836438

[8] Eutsler J, Harris MK, Williams LT, Cornejo OEJA, Organizations, Society. Accounting for partisanship and politicization: Employing Benford’s Law to examine misreporting of COVID-19 infection cases and deaths in the United States. 2023:101455.

[9] Ioannidis JP, Zonta F, Levitt MJEJoCI. Estimates of COVID‐19 deaths in Mainland China after abandoning zero COVID policy. 2023;53(4):e13956. doi: 10.1111/eci.13956 36691703 PMC10407651

[10] McMenamin M, Kolmer J, Djordjevic I, Campbell F, Laurenson-Schafer H, Abbate JL, et al. WHO Global Situational Alert System: a mixed methods multistage approach to identify country-level COVID-19 alerts. BMJ global health. 2023;8(7). doi: 10.1136/bmjgh-2023-012241 37495371 PMC10373705

[11] Bagi HM, Soleimanpour M, Abdollahi F, Soleimanpour HJPo. Evaluation of clinical outcomes of patients with mild symptoms of coronavirus disease 2019 (COVID-19) discharged from the emergency department. 2021;16(10):e0258697. doi: 10.1371/journal.pone.0258697 34673806 PMC8530279

[12] Coombs W.T., 2015. The value of communication during a crisis: Insights from strategic communication research. Business horizons, 58(2), pp.141–148.

[13] Wukich C., 2015. Social media use in emergency management. Journal of Emergency Management, 13(4), pp.281–294. doi: 10.5055/jem.2015.0242 26312654

[14] Von Muhlen M, Ohno-Machado LJJotAMIA. Reviewing social media use by clinicians. 2012;19(5):777–81. doi: 10.1136/amiajnl-2012-000990 22759618 PMC3422846

[15] Smailhodzic E, Hooijsma W, Boonstra A, Langley DJJBhsr. Social media use in healthcare: A systematic review of effects on patients and on their relationship with healthcare professionals. 2016;16(1):1–14. doi: 10.1186/s12913-016-1691-0 27562728 PMC5000484

[16] Ahmad AR, Murad HRJJomIr. The impact of social media on panic during the COVID-19 pandemic in Iraqi Kurdistan: online questionnaire study. 2020;22(5):e19556. doi: 10.2196/19556 32369026 PMC7238863

[17] Pagoto S, Waring ME, Xu RJJoMIR. A call for a public health agenda for social media research. 2019;21(12):e16661. 10.2196/16661 PMC6940852 31855185

[18] Chao M, Xue D, Liu T, Yang H, Hall BJJJoad. Media use and acute psychological outcomes during COVID-19 outbreak in China. 2020;74:102248. doi: 10.1016/j.janxdis.2020.102248 32505918 PMC7255752

[19] Li Y-J, Marga JJ, Cheung CM, Shen X-L, Lee MJAToH-CI. Health Misinformation on Social Media: A Systematic Literature Review and Future Research Directions. 2022;14(2):116–49.

[20] Li X, Liu QJJomIr. Social media use, eHealth literacy, disease knowledge, and preventive behaviors in the COVID-19 pandemic: Cross-sectional study on Chinese netizens. 2020;22(10):e19684. doi: 10.2196/19684 33006940 PMC7581310

[21] Wu G, Deng X, Liu B. Managing urban citizens’ panic levels and preventive behaviours during COVID-19 with pandemic information released by social media. Cities (London, England). 2022;120:103490. doi: 10.1016/j.cities.2021.103490 34642529 PMC8498698

[22] Demuyakor JJD, J. Social media, it C-pEpop. Social media and COVID-19 pandemic: enhancing panic or preventing it? 2021:211–22.

[23] Laranjo L, Arguel A, Neves AL, Gallagher AM, Kaplan R, Mortimer N, et al. The influence of social networking sites on health behavior change: a systematic review and meta-analysis. 2015;22(1):243–56. doi: 10.1136/amiajnl-2014-002841 25005606 PMC4433372

[24] Liu BF, Fraustino JD, Jin YJCR. Social media use during disasters: How information form and source influence intended behavioral responses. 2016;43(5):626–46.

[25] Liu HJBPH. Official social media and its impact on public behavior during the first wave of COVID-19 in China. 2022;22(1):1–13. doi: 10.1186/s12889-022-12803-y 35241057 PMC8893355

[26] Gamboa J, Lamb MM, de la Cruz P, Bull S, Olson DJM. Using social media to increase preventative behaviors against arboviral diseases: a pilot study among teens in the Dominican Republic. 2019;5. 10.21037/mhealth.2019.07.03 PMC6737439 31559275

[27] Kouzy R, Abi Jaoude J, Kraitem A, El Alam MB, Karam B, Adib E, et al. Coronavirus goes viral: quantifying the COVID-19 misinformation epidemic on Twitter. 2020;12(3). 10.7759/cureus.7255 PMC7152572 32292669

[28] Nwogwugwu DIJRAoMC, Communications R. Evaluation of research trends in social media crisis communication. 2023:438–59.

[29] Nicholls N, Yitbarek EJPo. Trust in social media and COVID-19 beliefs and behaviours. 2022;17(10):e0275969. doi: 10.1371/journal.pone.0275969 36227887 PMC9560499

[30] Olaoye A, Onyenankeya KJHPP. A systematic review of health communication strategies in Sub-Saharan Africa-2015-2022. 2023;13(1):10. doi: 10.34172/hpp.2023.02 37309431 PMC10257569

[31] Yassin A, AlOmari M, Al-Azzam S, Karasneh R, Abu-Ismail L, Soudah OJIjoehr. Impact of social media on public fear, adoption of precautionary behaviors, and compliance with health regulations during COVID-19 pandemic. 2022;32(9):2027–39. doi: 10.1080/09603123.2021.1935778 34097535

[32] Basch CH, Hillyer GC, Meleo-Erwin ZC, Jaime C, Mohlman J, Basch CEJJph, et al. Preventive behaviors conveyed on YouTube to mitigate transmission of COVID-19: cross-sectional study. 2020;6(2):e18807. doi: 10.2196/18807 32240096 PMC7124952

[33] Lin Y, Hu Z, Alias H, Wong LPJJomIr. Influence of mass and social media on psychobehavioral responses among medical students during the downward trend of COVID-19 in Fujian, China: cross-sectional study. 2020;22(7):e19982. doi: 10.2196/19982 32584779 PMC7373377

[34] Lu J, Wang X, Fei L, Chen G, Feng YJIT, People. Effects of social media empowerment on COVID-19 preventive behaviors in China. 2023.

[35] Cantwell O, Kushlev K. Anxiety talking: Does anxiety predict sharing information about COVID-19? 2021.

[36] Freiling I, Krause NM, Scheufele DA, Brossard DJNM, Society. Believing and sharing misinformation, fact-checks, and accurate information on social media: The role of anxiety during COVID-19. 2023;25(1):141–62. doi: 10.1177/14614448211011451 36620434 PMC9805917

[37] Kadam AB, Atre SRJJotm. Negative impact of social media panic during the COVID-19 outbreak in India. 2020;27(3):taaa057. doi: 10.1093/jtm/taaa057 32307545 PMC7188175

[38] Vaezi A, Javanmard SHJAbr. Infodemic and risk communication in the era of CoV-19. 2020;9. 10.4103/abr.abr_47_20 PMC7147513 32309248

[39] Clemente-Suárez VJ, Navarro-Jiménez E, Simón-Sanjurjo JA, Beltran-Velasco AI, Laborde-Cárdenas CC, Benitez-Agudelo JC, et al. Mis–dis information in COVID-19 health crisis: A Narrative review. 2022;19(9):5321. doi: 10.3390/ijerph19095321 35564714 PMC9101334

[40] Brainard J, Hunter PRJS. Misinformation making a disease outbreak worse: outcomes compared for influenza, monkeypox, and norovirus. 2020;96(4):365–74. doi: 10.1177/0037549719885021 34285423 PMC8282656

[41] Garfin DR, Silver RC, Holman EAJHp. The novel coronavirus (COVID-2019) outbreak: Amplification of public health consequences by media exposure. 2020;39(5):355. doi: 10.1037/hea0000875 32202824 PMC7735659

[42] Mertens G, Gerritsen L, Duijndam S, Salemink E, Engelhard IMJJoad. Fear of the coronavirus (COVID-19): Predictors in an online study conducted in March 2020. 2020;74:102258. 10.1016/j.janxdis.2020.102258 PMC7286280 32569905

[43] Limaye RJ, Sauer M, Ali J, Bernstein J, Wahl B, Barnhill A, et al. Building trust while influencing online COVID-19 content in the social media world. 2020;2(6):e277–e8. doi: 10.1016/S2589-7500(20)30084-4 32322814 PMC7173823

[44] Basch CH, Fera J, Pellicane A, Basch CEJI, Disease, Health. Handwashing videos on TikTok during the COVID-19 pandemic: Potential for disease prevention and health promotion. 2022;27(1):31–7. 10.1016/j.idh.2021.09.039 PMC8495082 34690108

[45] Breza E, Stanford FC, Alsan M, Alsan B, Banerjee A, Chandrasekhar AG, et al. Effects of a large-scale social media advertising campaign on holiday travel and COVID-19 infections: a cluster randomized controlled trial. 2021;27(9):1622–8. doi: 10.1038/s41591-021-01487-3 34413518 PMC8440209

[46] Smail EJ, Livingston T, Wolach A, Cenko E, Kaufmann CN, Manini TMJJFR. Media Consumption and COVID-19–Related Precautionary Behaviors During the Early Pandemic: Survey Study of Older Adults. 2023;7:e46230. doi: 10.2196/46230 37213166 PMC10242469

[47] Solnick RE, Chao G, Ross RD, Kraft‐Todd GT, Kocher KEJAEM. Emergency physicians and personal narratives improve the perceived effectiveness of COVID‐19 public health recommendations on social media: A randomized experiment. 2021;28(2):172–83. doi: 10.1111/acem.14188 33263357 PMC7753341

[48] Wang H, Li Y, Hutch M, Naidech A, Luo YJJomIr. Using tweets to understand how COVID-19–Related health beliefs are affected in the age of social media: Twitter data analysis study. 2021;23(2):e26302. doi: 10.2196/26302 33529155 PMC7901597

[49] Tsoy D, Godinic D, Tong Q, Obrenovic B, Khudaykulov A, Kurpayanidi KJS. Impact of social media, Extended Parallel Process Model (EPPM) on the intention to stay at home during the COVID-19 pandemic. 2022;14(12):7192.

[50] Liu PLJSS, Medicine. COVID-19 information on social media and preventive behaviors: Managing the pandemic through personal responsibility. 2021;277:113928. 10.1016/j.socscimed.2021.113928 PMC8040317 33865093

[51] Niu Z, Qin Z, Hu P, Wang TJHC. Health beliefs, trust in media sources, health literacy, and preventive behaviors among high-risk Chinese for COVID-19. 2022;37(8):1004–12. doi: 10.1080/10410236.2021.1880684 33557620

[52] Wu G, Deng X, Liu BJC. Managing urban citizens’ panic levels and preventive behaviours during COVID-19 with pandemic information released by social media. 2022;120:103490. doi: 10.1016/j.cities.2021.103490 34642529 PMC8498698

[53] Yu J, Gorman BS, Springer CMJITJoHCO, Provision, Financing. Source of Information on Intentions to Adopt Protective Behaviours During COVID-19 in China. 2022;59:00469580221090411. 10.1177/00469580221090411 PMC9082741 35506690

[54] Mat Dawi N, Namazi H, Maresova PJFiP. Predictors of COVID-19 preventive behavior adoption intention in Malaysia. 2021;12:616749. doi: 10.3389/fpsyg.2021.616749 34093307 PMC8172794

[55] Mohammed F, Al-Kumaim NH, Alzahrani AI, Fazea YJIjoer, health p. The impact of social media shared health content on protective behavior against COVID-19. 2023;20(3):1775. doi: 10.3390/ijerph20031775 36767142 PMC9914293

[56] Zhang SX, Graf-Vlachy L, Looi KH, Su R, Li JJE, Infection. Social media use as a predictor of handwashing during a pandemic: evidence from COVID-19 in Malaysia. 2020;148:e261. 10.1017/S0950268820002575 PMC7653491 33092675

[57] Choi D-H, Noh G-YJSO. The Impact of Social Media on Preventive Behavior During the COVID-19 Outbreak in South Korea: The Roles of Social Norms and Self-Efficacy. 2023;13(3):21582440231184969. doi: 10.1177/21582440231184969 37456583 PMC10333550

[58] Lee J, Kim K, Park G, Cha NJT, Informatics. The role of online news and social media in preventive action in times of infodemic from a social capital perspective: The case of the COVID-19 pandemic in South Korea. 2021;64:101691. 10.1016/j.tele.2021.101691 PMC9759650 36567818

[59] Yu S, Abbas J, Draghici A, Negulescu OH, Ain NUJFiP. Social media application as a new paradigm for business communication: the role of COVID-19 knowledge, social distancing, and preventive attitudes. 2022;13:903082. doi: 10.3389/fpsyg.2022.903082 35664180 PMC9160995

[60] Hernández-García I, Giménez-Júlvez TJIJoER, Health P. Characteristics of YouTube videos in Spanish on how to prevent COVID-19. 2020;17(13):4671. doi: 10.3390/ijerph17134671 32610523 PMC7370194

[61] Abuhashesh MY, Al-Dmour H, Masa’deh Re, Salman A, Al-Dmour R, Boguszewicz-Kreft M, et al., editors. The role of social media in raising public health awareness during the pandemic COVID-19: an International Comparative Study. Informatics; 2021: MDPI.

[62] Wardani EM, Bistara DN, Setiyowati EJSJIK. The influence of social media about COVID-19 on handwashing behavior, mask wearing and physical distancing of Indonesian students. 2020;9(2):1338–45.

[63] O’Brien N, Vijaykumar S, Craig M, Land E, Jocol SMA, Mendoza XGB, et al. A pre-and post-intervention study testing the effect of exposure to languageless animated images communicating COVID-19 preventive behaviours on behavioural intentions and beliefs of Guatemalan adults. 2022;12. 10.7189/jogh.12.05018 PMC9304925 35862262

[64] Suzuki T, Yamamoto H, Ogawa Y, Umetani RJH, Communications SS. Effects of media on preventive behaviour during the COVID-19 pandemic. 2023;10(1):1–8. doi: 10.1057/s41599-023-01554-9 36818040 PMC9926457

[65] Duong HT, Nguyen LTV, Julian McFarlane S, Nguyen HT, Nguyen KTJHC. Preventing the COVID-19 outbreak in Vietnam: Social media campaign exposure and the role of interpersonal communication. 2023;38(2):394–401. doi: 10.1080/10410236.2021.1953729 34278892

[66] Islam MM, Islam MM, Ahmed F, Rumana ASJSNA, Mining. Creative social media use for Covid-19 prevention in Bangladesh: a structural equation modeling approach. 2021;11:1–14. 10.1007/s13278-021-00744-0 PMC8036010 33868509

[67] Sadore AA, Handiso DW, Wontamo TE, Mekango DE, Moges SJDM, Preparedness PH. Influence of social media use on practice of COVID-19 preventive measures among Ethiopian residents: an online cross-sectional study. 2022;16(6):2546–51. 10.1017/dmp.2021.184 PMC8314057 34099090

[68] Graffigna G, Bosio C, Savarese M, Barello M, Barello SJFip. “# I-Am-Engaged”: Conceptualization and First Implementation of a Multi-Actor Participatory, Co-designed Social Media Campaign to Raise Italians Citizens’ Engagement in Preventing the Spread of COVID-19 Virus. 2020;11:567101. doi: 10.3389/fpsyg.2020.567101 33250811 PMC7674954

[69] Okpara CV, Anselm AU, Felix TO, Omowale A, Gever VCJHpi. The moderating role of colour in modelling the effectiveness of COVID-19 YouTube animated cartoons on the health behaviour of social media users in Nigeria. 2021;36(6):1599–609. doi: 10.1093/heapro/daab001 33729511 PMC7989244

[70] Alrasheed M, Alrasheed S, Alqahtani ASJSJoHSR. Impact of social media exposure on risk perceptions, mental health outcomes, and preventive behaviors during the COVID-19 pandemic in Saudi Arabia. 2022;2(3):107–13.

[71] Bridgman A, Merkley E, Loewen PJ, Owen T, Ruths D, Teichmann L, et al. The causes and consequences of COVID-19 misperceptions: Understanding the role of news and social media. 2020;1(3).

[72] Iyamu I, Apantaku G, Yesufu Z, Oladele EA, Eboreime E, Afirima B, et al. Is social media, as a main source of information on COVID-19, associated with perceived effectiveness of face mask use? Findings from six sub-Saharan African countries. 2022;29(3):86–96. doi: 10.1177/17579759211065489 35081834 PMC9607963

[73] Vandormael A, Adam M, Greuel M, Gates J, Favaretti C, Hachaturyan V, et al. The effect of a wordless, animated, social media video intervention on COVID-19 prevention: online randomized controlled trial. 2021;7(7):e29060. doi: 10.2196/29060 34174778 PMC8317990

[74] Mwaura J, Carter V, Kubheka BZJAJoPHC, Medicine F. Social media health promotion in South Africa: Opportunities and challenges. 2020;12(1):1–7. 10.4102/phcfm.v12i1.2389 PMC7433217 32787400

[75] Rubyan-Ling DJD-c-a-h-s-bp. Briefing Paper: Diaspora communications and health seeking behavior in the time of Ebola: findings from the Sierra Leonean community in London: Ebola Response Anthropology Platform; 2015 [Available from: http://www.Ebola-anthropology.Net/wpcontent/uploads/2015/11.

[76] Merchant RM, Lurie NJJ. Social media and emergency preparedness in response to novel coronavirus. 2020;323(20):2011–2. doi: 10.1001/jama.2020.4469 32202611

[77] Yousuf H, Corbin J, Sweep G, Hofstra M, Scherder E, van Gorp E, et al. Association of a public health campaign about coronavirus disease 2019 promoted by news media and a social influencer with self-reported personal hygiene and physical distancing in the Netherlands. 2020;3(7):e2014323-e. doi: 10.1001/jamanetworkopen.2020.14323 32639569 PMC7344381

[78] Choo EK, Ranney ML, Chan TM, Trueger NS, Walsh AE, Tegtmeyer K, et al. Twitter as a tool for communication and knowledge exchange in academic medicine: a guide for skeptics and novices. 2015;37(5):411–6. doi: 10.3109/0142159X.2014.993371 25523012

[79] Hopfer S, Fields EJ, Lu Y, Ramakrishnan G, Grover T, Bai Q, et al. The social amplification and attenuation of COVID-19 risk perception shaping mask wearing behavior: a longitudinal twitter analysis. 2021;16(9):e0257428. doi: 10.1371/journal.pone.0257428 34555060 PMC8460003

[80] Lee JL, DeCamp M, Dredze M, Chisolm MS, Berger ZDJJomIr. What are health-related users tweeting? A qualitative content analysis of health-related users and their messages on twitter. 2014;16(10):e237. doi: 10.2196/jmir.3765 25591063 PMC4296104

[81] Sahni H, Sharma HJIJoAM. Role of social media during the COVID-19 pandemic: Beneficial, destructive, or reconstructive? 2020;6(2):70–5.

[82] Schillinger D, Chittamuru D, Ramírez ASJAjoph. From “infodemics” to health promotion: a novel framework for the role of social media in public health. 2020;110(9):1393–6. 10.2105/AJPH.2020.305746 PMC7427212 32552021

[83] Van der Meer TG, Jin YJHC. Seeking formula for misinformation treatment in public health crises: The effects of corrective information type and source. 2020;35(5):560–75. doi: 10.1080/10410236.2019.1573295 30761917

[84] Joseph AM, Fernandez V, Kritzman S, Eaddy I, Cook OM, Lambros S, et al. COVID-19 misinformation on social media: a scoping review. 2022;14(4). 10.7759/cureus.24601 PMC9148617 35664409

[85] Peng Y, Pei C, Zheng Y, Wang J, Zhang K, Zheng Z, et al. Knowledge, attitude and practice associated with COVID-19 among university students: A cross-sectional survey in China. 2020. 10.1186/s12889-020-09392-z PMC7447607 32847554

[86] Ssebuufu R, Sikakulya FK, Binezero SM, Wasingya L, Nganza SK, Ibrahim B, et al. Awareness, knowledge, attitude and practice towards measures for prevention of the spread of COVID-19 in the Ugandans: A nationwide online cross-sectional Survey. 2020:2020.05. 05.20092247. 10.3389/fpubh.2020.618731 PMC7793670 33425842

[87] Allington D, Duffy B, Wessely S, Dhavan N, Rubin JJPm. Health-protective behaviour, social media usage and conspiracy belief during the COVID-19 public health emergency. 2021;51(10):1763–9. doi: 10.1017/S003329172000224X 32513320 PMC7298098

[88] Boyd NM, Martin ECJAT, Praxis. Sense of community responsibility at the forefront of crisis management. 2022;44(1):71–83.

[89] Tsoy D, Tirasawasdichai T, Kurpayanidi KIJIJoMS, Administration B. Role of social media in shaping public risk perception during COVID-19 pandemic: A theoretical review. 2021;7(2):35–41.

[90] Wilson EA, Makoul G, Bojarski EA, Bailey SC, Waite KR, Rapp DN, et al. Comparative analysis of print and multimedia health materials: a review of the literature. 2012;89(1):7–14. doi: 10.1016/j.pec.2012.06.007 22770949

