# Supplementary material for: The role of social media on COVID-19 preventive behaviors worldwide, systematic review
Source: PLoS One. 2024 Jul 10;19(7):e0306284. doi: 10.1371/journal.pone.0306284 (PMC11236194; doi:10.1371/journal.pone.0306284)
Supplement: S1 Table — (PDF) [file pone.0306284.s002.pdf]

Table 1. The role of social media on COVID-19 preventive behaviors worldwide, systematic review

First author/ Pub year	Study design	Country	Sample size	Social media and COVID-19 preventive behaviors	Quality score
Hernández-García I et al. (54)/2020	cross-sectional study	Spain	129	videos to be viewed in order to obtain reliable information on hand washing practice.	7
Basch CH, et al. (38)/2022	cross-sectional study	USA	100	Wash your hands accounted for 93.3% of the total views. Coverage of the important steps involved in handwashing, such as drying hands, was minimal as was relevant background information.	6
Abuhashesh MY etal.(55)/2021	cross-sectional study	Poland and Jordan	1149	positive relationships between the components of a social media campaign, public health awareness, and behavioral change during COVID-19.	8
Smail EJ et al.(40)/2023	cross-sectional study	USA	1057	Increasing social media use was associated with engagement in more COVID-19 precautionary behaviors.	7
Zhang SX,et al (50)/2020	cross-sectional study	Malaysia	674	More time spent on social media was positively associated with hand washing for males with three or more children. However, for males without children, social media use	7

				was negatively associated with 5handwashing.	
Wardani EM, etal/2020(56)	Experimental	Indonesia	5400	Social media is effectively used to provide health education because it can increase student knowledge and influence behavior in preventing covid-19 transmission.	9
O'Brien N, et al/2022(57)	Experimental	Guatemala	308	GIF exposure significantly improved participants' self-efficacy, intention and belief on hand washing behavior.	8
Yu J, et al(47) /2022	cross-sectional study	China	122	People who trust in informal information from social media and interpersonal communication would be more likely to adopt mask wearing and hand washing protective behaviors.	5
Suzuki T, et al (58) (2021)	cross-sectional study	Japan	987	social media had stronger predictive power to change attitude through time.	6
Breza E ,et al. (39)/2021	Experimental	USA	410	Social media messages recorded by health professionals before the winter holidays in the United States led to a significant reduction in holiday travel and subsequent COVID-19 infections.	6
Yassin A,et al (34)(2022)	cross-sectional study	Jordan	827	The variables that were significantly associated with higher levels of precautionary behaviors were knowledge, feeling of fear,	8

				and following medical pages on social media.	
Niu Z ,et al/2021(45)	cross-sectional study	China	464	The results suggested that the vulnerable populations' engagement in coronavirus-related preventive behaviors were significantly associated with barriers, benefits, self-efficacy, trust in doctors' social media, and trust in TV for COVID-19-related information. Besides, barriers, benefits, self-efficacy, trust in doctors' social media, and trust in TV mediated the effects of health literacy on preventive behaviors.	7
Duong HT, et al (59)/2021	cross-sectional study	Vietnam	360	Results indicated that interpersonal communication mediated the effect of social media campaign exposure on intentions to stay at home.	7
Tsoy D,et al (43)/2022	cross-sectional study	USA	306	social media would have a positive impact on staying at home intentions. But if users instead perceived messages as being fabricated, amplified, or false, the alert would stay dangerously low.	8
Islam MM,et al (60) /2021	cross-sectional study	Banglade sh	265	Creative social media increase knowledge on COVID-19 prevention measures by using seven online activities which are awareness of Covid-19 spread, wearing the mask, hand washing,	5

				precaution before touching the face, nose and eyes, avoiding gathering, social distances, and knowing Covid-19 symptoms. These activities are exhibited in reading essays/writings, seeing photos and flyers, watching videos posting writings, pictures, flyers and videos on social media platforms like Facebook, WhatsApp and YouTube.	
Wang H,et al(42)/2021	cross-sectional study	USA	500	The excessive information disseminated on social media platforms and other sources is closely related to the dynamics of the general public's health beliefs.	6
Wu G, et al (46) /2020	cross-sectional study	China	592	Social media information sources, unofficial social media caused both Wuhan and non-Wuhan urban citizens to have higher levels of panic than official media but had no significant impacts on their preventive behaviors.	7
Lu J,et al (37)/2023	cross-sectional study	China	739	Social media empowers individuals in terms of knowledge seeking, knowledge sharing, socializing and entertainment to promote preventive behaviors at the individual level by increasing each person's perception of collective efficacy and social cohesion.	7

Sadore AA, et al (61)/2021	cross-sectional study	Ethiopia	372	Study participants who had good use of social media to get COVID-19–related information were 9.5 times more engaged in COVID-19 preventive practices than non-users. The study participants who had a high-risk perception of COVID-19 were 2.6 times more engaged in COVID-19 practices compared with study participants who had a low-risk perception of COVID-19.	6
Graffigna G, et al (62)/2020	cross-sectional study	Italy	976	10% of the audience showed an active engagement with the campaign, by expressing likes, by writing comments on, or sharing its contents on Facebook and LinkedIn. Facebook generally appears a more suitable platform for engaging with the audience and as a means to convey public health information in a lively manner.	8
Choi D-H, et al, /2023 (51)	cross-sectional study	Korea	1500	Social media use is positively associated with social norms, which may have a positive relationship with COVID-19 preventive behavior.	6
Mohammed F,(49)/2023	cross-sectional study	Malaysia	488	The findings showed that perceived risk, e-health literacy, public awareness, and health experts' participation influence public protective behavior when using	5

				social media to share COVID-19-relevant content.	
Okpara CV,et al (63)/2021	cross-sectional study	Nigeria	470	The result showed that recall of messages theme in COVID-19 YouTube animated cartoons significantly predicts health behavior of social media users.	6
Yu S,et al (53) /2022	cross-sectional study	Pakistan	348	The findings approve that attitudes toward social media use in the pandemic have positively mediated the relation between distancing and practices for social media use amid the crisis of COVID-19.	5
Solnick RE, et al (41)/2021	experimental study	USA	2007	The public health messages delivered by physicians and personal messages elicited stronger emotions, greater changes in attitudes, and an increased willingness to disseminate the message than when federal officials delivered impersonal messages.	6
Alrasheed M,et al (64)/2022	cross-sectional study	Saudi Arabia	1500	93.2% of participants use social media for COVID-19 related information. High social media exposure was significantly associated with higher risks of anxiety, depression, and higher levels of COVID-19 risk perception. However, social media has no significant impact on the adoption of preventive behavior.	7

Mat Dawi N,et al /2021(48)	cross-sectional study	Malaysia	404	perception of e-government information and services and perception of social media were found to be significant predictors of attitude toward preventive behavior.	6
Bridgman A,et al (65)/2020	cross-sectional study	Canada	500	Exposure to social media is associated with misperceptions regarding basic facts about COVID-19 while the inverse is true for news media. These misperceptions are in turn associated with lower compliance with social distancing measures.	5
Lee J,et al/2021(52)	cross-sectional study	South Korea	1000	The results reveal that the perceived characteristics of online news and social media influence preventive actions through the trust in citizens or in government.	7
Iyamu I,(66) /2021	cross-sectional study	SSA	1988	The respondents who used social media were more likely to agree that face masks were effective compared with those who did not.	7
Liu PLJSS,et al (44)/2021	cross-sectional study	China	511	Results indicated that personal responsibility partially mediated the relationship between COVID-19 information consumption on social media and preventive behaviors.	6
Vandormael A, et al (67)/2021	RCT	USA, & Europe	14,482	Short, wordless, animated videos, distributed by health authorities via social media, may be an effective	10

				pathway for rapid global health communication during health crises.	
